# Molecular size dependence on achievable resolution from XFEL single-particle 3D reconstruction

**DOI:** 10.1063/4.0000175

**Published:** 2023-03-17

**Authors:** Miki Nakano, Osamu Miyashita, Florence Tama

**Affiliations:** 1 RIKEN Center for Computational Science, 6-7-1, Minatojima-minami-machi, Chuo-ku, Kobe, Hyogo 650-0047, Japan; 2 Department of Physics, Graduate School of Science, Nagoya University, Furo-cho, Chikusa-ku, Nagoya, Aichi 464-8602, Japan; 3 Institute of Transformative Bio-Molecules, Nagoya University, Furo-cho, Chikusa-ku, Nagoya, Aichi 464-8602, Japan

## Abstract

Single-particle analysis using x-ray free-electron lasers (XFELs) is a novel method for obtaining structural information of samples in a state close to nature. In particular, it is suitable for observing the inner structure of large biomolecules by taking advantage of the high transmittance of x-rays. However, systematic studies on the resolution achievable for large molecules are lacking. In this study, the molecular size dependence of the resolution of a three-dimensional (3D) structure resulting from XFEL single-particle reconstruction is evaluated using synthetic data. Evidently, 3D structures of larger molecules can be restored with higher detail (defined relative to the molecular sizes) than smaller ones; however, reconstruction with high absolute resolution (defined in nm^−1^) is challenging. Our results provide useful information for the experimental design of 3D structure reconstruction using coherent x-ray diffraction patterns of single-particles.

## I. INTRODUCTION

The highly coherent light source of x-ray free-electron lasers (XFELs) enables the collection of diffraction data from molecules in a state close to nature without crystallization.^1–8^ Additionally, a short and intense femtosecond pulse allows capturing the momentary structure of the target sample before destruction.^1,4,9–11^ This “diffraction before destruction” allows observing the sample without cryogenic conditions and, thus, is useful for studying biomolecules.

This light source enables observing the inner structures of large molecules, more than several hundred nanometers, because the high transmittance of x-rays reduces multiple scattering events, which is a significant problem in cryo-electron microscopy.^7,12–16^ Additionally, a larger x-ray cross section of a larger molecule is attractive because it provides more scattered photons, thus reducing the influence of shot noise.

However, to restore molecular structures with high resolution, capturing sufficient scattered photons in the wide-angle scattering region is more critical than the total number of detected photons. In general, the detected photon count decreases rapidly with the scattering angle.^17^ Whether larger molecules can provide scattered photons in the wide-angle scattering regions sufficient for retrieving the high-resolution structure is unclear. In addition, the pixel sizes of the diffraction patterns must be smaller for larger molecules to maintain a sufficient oversampling ratio for successful phase recovery,^18,19^ thus effectively reducing the photon counts captured on each pixel. Understanding the impact of pixel size on the diffraction patterns from experiments is crucial for reconstructing the molecular structures of the target sample at the desired resolution.

Experimental structural reconstructions from XFEL single-particle diffraction patterns have been performed for viruses of various sizes.^7,12,13^ A compilation of these results reveals that a smaller virus structure was reconstructed with higher resolution: 125-nm resolution for Giant Mimivirus with 450-nm diameter,^12^ 28-nm resolution for Melbournevirus with 230-nm diameter,^13^ and less than 9 nm resolution for PR772 with 70-nm diameter.^7^ However, the number of diffraction patterns used for reconstruction differed among these studies; additionally, the XFEL pulse intensities and photon energies were different. Therefore, the size-dependence of the final resolution cannot be deduced from these studies.

The requirements for successful three-dimensional (3D) structure reconstruction have been discussed through theoretical simulations.^20–24^ In particular, analytical formulas to estimate the number of diffraction patterns required to reach a certain resolution for a given molecule have been proposed.^24^ In this study, we examined the effect of the target molecular size on the expected resolutions of the structures from XFEL single-particle analysis by performing simulations that go through the procedures for 3D reconstruction, starting from angle estimation, diffraction pattern assembly, and phase retrieval. Five biological molecules of different sizes, ranging from approximately 20 to 120 nm, with asymmetric structures, were selected. Simulated diffraction patterns were created for these molecules assuming the same experimental setup. The properties of the 3D diffraction intensity distribution reconstructed from two-dimensional (2D) patterns, angle estimation accuracy, and resolution of the restored molecular structures were evaluated. Our results provide useful information for the experimental design, such as the beam intensity, requirement of the number of diffraction patterns, detector settings, and sample selection through the assessment of the achievable resolutions of the tested target samples.

## II. MATERIALS AND METHODS

### A. Simulated diffraction patterns

**FIG. 1.**
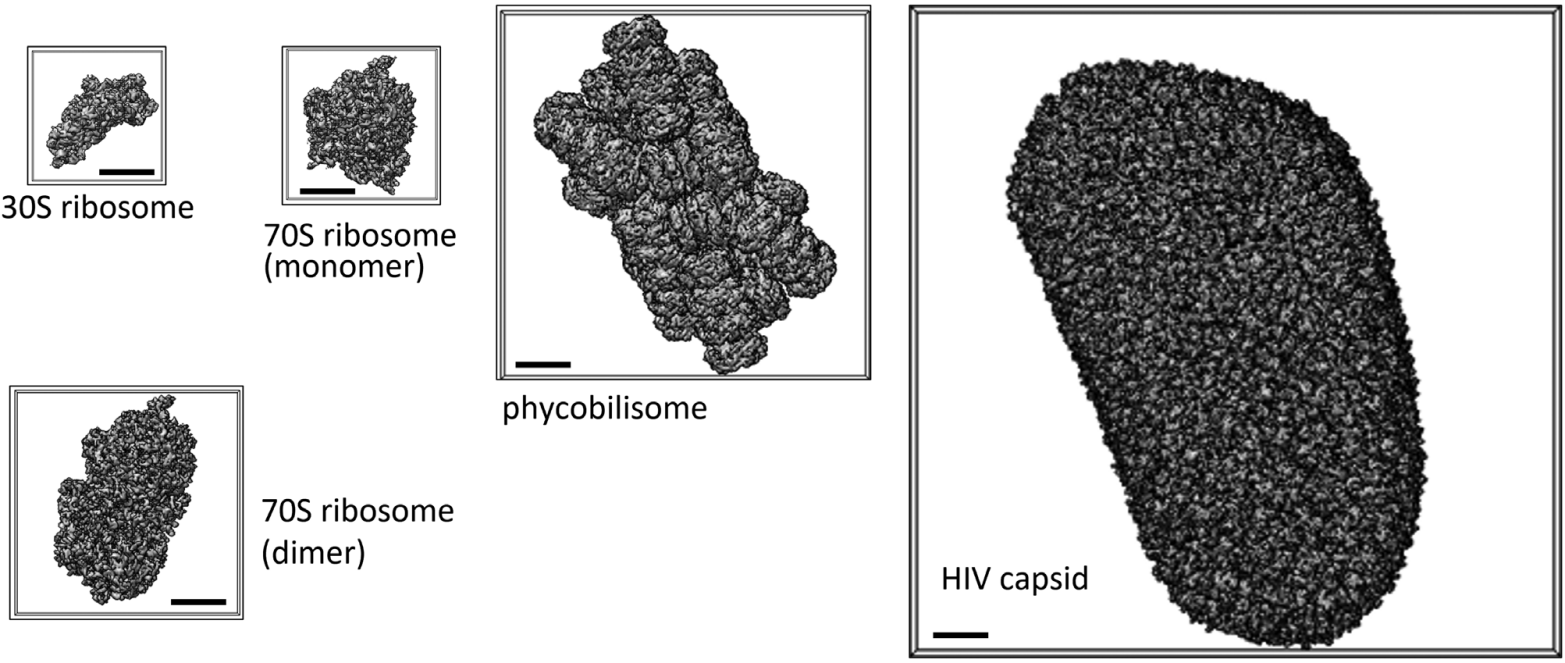
Molecular structures and sizes used in this study. All scale bar lengths in the figure correspond to 10 nm.

Herein, five biomolecules of different sizes were selected: a 30S ribosome (PDB ID: 4nxn),^25^ a 70S ribosome monomer, a dimer (PDB ID: 4v67),^26^ a phycobilisome (PDB ID: 5y6p),^27^ and a human immunodeficiency virus (HIV) capsid (PDB ID: 3j3q).^28^ Figure 1 shows electron density maps of the molecules simulated from the atomic structures. These molecules ranged in size from approximately 20 to 120 nm.

The diffraction patterns of these molecules were generated by assuming XFEL pulse irradiations with a wavelength of 0.4 nm. The size of the simulated diffraction patterns was 591^2^ pixels, with the edge corresponding to 1.25 nm^−1^ in reciprocal space. Two beam intensities were simulated: 5.0 × 10^13^ and 5.0 × 10^12^ photons/*μ*m^2^, which are referred to as “strong” and “medium,” respectively; the medium beam intensity was close to that achieved at recent XFEL facilities.^29–32^ The diffraction pattern sets were prepared with different numbers of patterns: ten thousand (10 K) and one hundred thousand (100 K). Recently, Euro-XFEL achieved megahertz repetition XFEL pulse and succeeded in obtaining more than 1 × 10^6^ single hit patterns.^19,33^ Therefore, these numbers of diffraction patterns would be achievable with the development of XFEL facilities.

To prepare the simulated diffraction patterns, 3D diffraction intensity distributions, *F_answer_*, were created by performing Fourier transform on the 3D electron density maps created from the PDB structures. The 2D diffraction patterns were created by slicing the *F_answer_* considering Ewald curvature. Poisson noise was added, such that the average photon count for each pixel corresponds to the count obtained using the assumed beam intensity.^34^ We developed the scripts for diffraction pattern creation based on Scipion, a widely used cryo-EM image processing package.^35^ Although our method is simple compared to the method calculating the scattered photons directly used for other simulation frameworks for XFEL single-particle analysis,^36,37^ it should provide sufficient evidence to evaluate the molecular size dependence of this experiment.

**FIG. 2.**
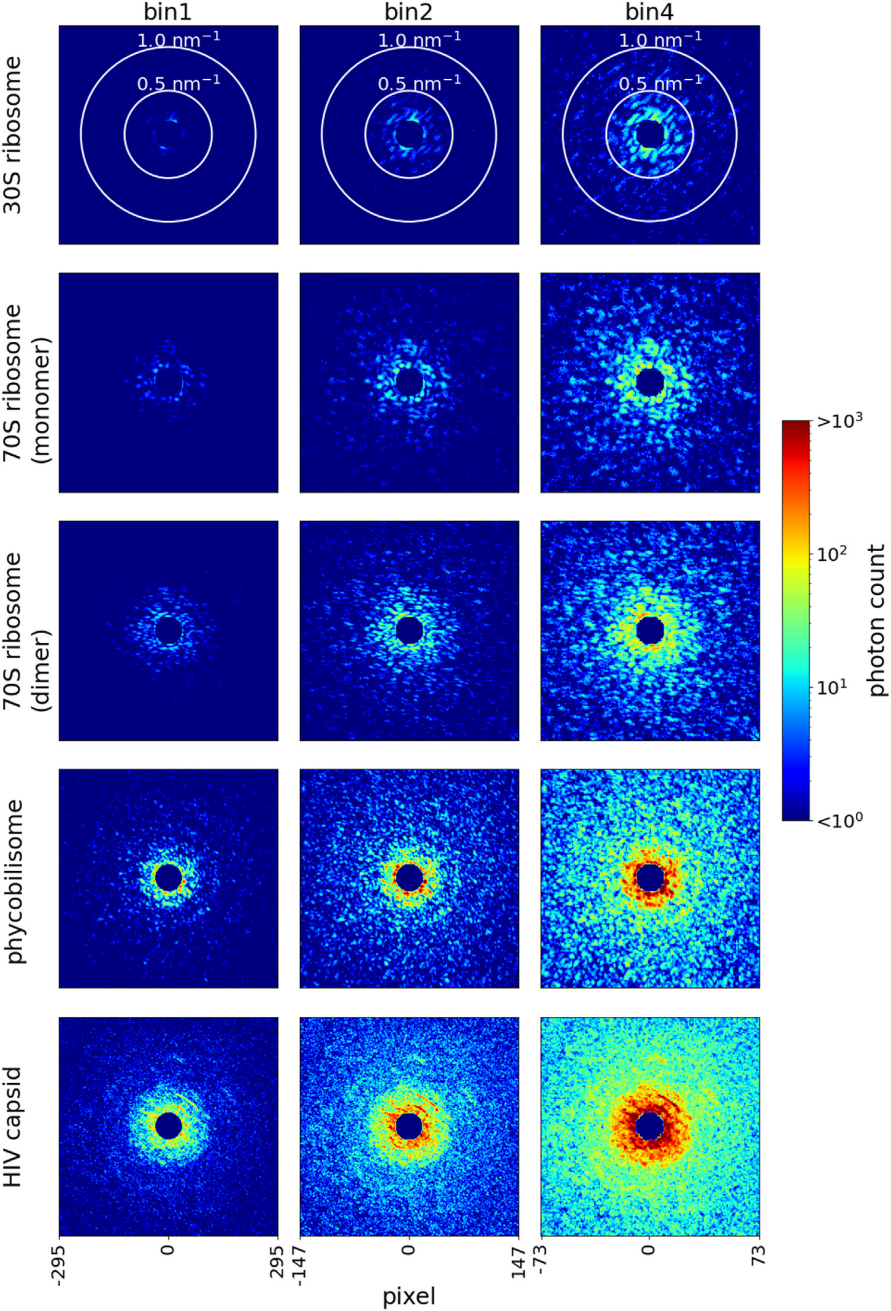
Simulated diffraction pattern samples created with strong beam intensity. The detector edge corresponds to 1.25 nm^−1^. Original diffraction patterns (bin1) consist of 591^2^ pixels. These diffraction patterns are binned to 2 × 2 pixels or 4 × 4 pixels (bin2 or bin4, respectively). The central regions of diffraction patterns are masked with a radius of 0.156 nm^−1^ by assuming the beam stopper.

The central region of the diffraction patterns was masked with a radius of 0.156 nm^−1^. Finally, each diffraction pattern was binned to 2 × 2 pixels (a factor of two in both dimensions) or 4 × 4 pixels (a factor of four in both dimensions). Hereafter, the results without binning are referred to as bin1, those binned by a factor of two in both dimensions as bin2, and those binned by a factor of four in both dimensions as bin4. Figure 2 shows examples of diffraction patterns. Note that binned pixels can capture more photons and reduce the contribution of shot noise.

The pixel size in reciprocal space Δ*q* is related to the oversampling ratio. An oversampled diffraction pattern, sampled with a finer sampling spacing than the Nyquist rate, is essential to restore the phase information lost on the detector.^16,38^ To compare the target molecules with different sizes, we use the linear oversampling ratio σ_*L*_, which is defined as 1/(*L*Δ*q*), where *L* denotes the molecular size. Because the molecular size is different in each direction, the exact σ_*L*_ values cannot be defined. Instead, the box size as shown in Fig. 1 was defined as *L*. Table I summarizes the Δ*q* and σ_*L*_ values for each binned pattern for each molecule. By definition, σ_*L*_ decreases with increasing molecular size for the same Δ*q*.

**TABLE I.** Pixel size of diffraction patterns and linear oversampling ratios for each molecule.

Binning factor		1 × 1 (bin1)	2 × 2 (bin2)	4 × 4 (bin4)	Photon counts/diffraction pattern^a^
Number of pixels		591 × 591	295 × 295	147 × 147	
Pixel size (Δ*q*^2^) (nm^−2^)		0.0042 × 0.0042	0.0085 × 0.0085	0.017 × 0.017	
Molecule	Size (*L*) (nm)	Linear oversampling ratio (σ_*L*_)			
30S ribosome	24.8	9.5	4.8	2.4	2.37 × 10^4^
70S ribosome (monomer)	28.4	8.3	4.2	2.1	6.37 × 10^4^
70S ribosome (dimer)	42.0	5.6	2.8	1.4	1.28 × 10^5^
Phycobilisome	68.4	3.5	1.7	0.9	4.52 × 10^5^
HIV capsid	118.0	2.0	1.0	0.5	1.11 × 10^6^

^a^ Average number of scattered photons among 10 K masked diffraction patterns without binning created with strong beam intensity.

In theory, to restore the phase information of *N*-dimensional objects, σ_*L*_ should be at least 2^1/*N*^,^39^ and a larger value is required for noisy and complex objects. Our previous study reported that σ_*L*_ ≥ 4 provides a good restoration of the molecular structure from the 3D diffraction intensity distribution.^34^ In this study, the minimum value of σ_*L*_ = 2, which was the condition for the unbinned pattern of HIV capsid, was considered. The binned diffraction patterns of HIV capsid could not be used for phase retrieval because the oversampling ratios were insufficient.

### B. Reconstruction of 3D diffraction intensity distribution from 2D diffraction patterns

In single-particle analysis using XFELs, the 3D diffraction intensity distribution is reconstructed by assembling the 2D diffraction patterns. This process requires interpolation to fill the gaps between the assembled 2D patterns in the 3D volume. To determine suitable interpolation parameters, the 3D structure factor intensity distribution was reconstructed from the simulated diffraction patterns by specifying the *true* angles used for the diffraction pattern creation, *F_true_angle_*.

To estimate the diffraction intensities of the voxels between the patterns, a weight function based on the Kaiser–Bessel window *w*(α, η; *d*) was used, defined as follows:^34,40^

$$w\left(d\right)=\frac{\xi \left(\alpha ,\eta \right)}{I_{0}\left(\pi \alpha \right)}I_{0}\left\{\pi \alpha \sqrt{1-{\left(\frac{2d-\eta }{\eta -1}-1\right)}^{2}}\right\},\;0\leq d\leq \eta .$$ (1)

I_0_ is the zeroth-order modified Bessel function, and ξ(α,η) is the normalization factor determined by α and η. The value of *w*(α, η; *d*) depends on the distance *d* between the center of the voxel, where the diffraction intensity is calculated, and the exact position mapped from the 2D pattern to the reconstructed 3D structure. Here, η determines the cutoff for interpolation length, and α determines the rate of decrease in the weight function. With a large α, *w*(*d*) decreases rapidly.

Herein, the α and η values were optimized by evaluating the spherical correlation functions between *F_answer_* and *F_true_angle_* defined by Eq. (2) as follows:

$$\mathrm{correlation}(q)=\frac{\sqrt{\sum_{i\in q}\left(x_{i}-\bar{x_{q}}\right)\left(y_{i}-\bar{y_{q}}\right)}}{\sqrt{\sum_{i\in q}{\left(x_{i}-\bar{x_{q}}\right)}^{2}}\sqrt{\sum_{i\in q}{\left(y_{i}-\bar{y_{q}}\right)}^{2}}},$$ (2)

where *q* is the spatial frequency defined as 2sin*θ*/*λ*, where *θ* is the half of the scattering angle and *λ* is the wavelength of the XFEL pulse. *x_i_* and *y_i_* represent the diffraction intensities in voxel *i* within shells of *q* in *F_answer_* and *F_true_angle_*, respectively. $\bar{x_{q}}$ and $\bar{y_{q}}$ represent the average diffraction intensities of the same shell. The optimized α and η values are listed in Table S1. A combination of small α and large η averages the diffraction intensity over distant pixels. This combination can effectively reduce shot noise when the number of photons in each pixel is low. On the other hand, this combination makes diffraction pattern blur. Therefore, a large α and small η combination is suitable for reconstruction from the diffraction patterns created by the strong beam intensity of larger molecules. Similarly, binned diffraction patterns have large photon counts, and large α and small η were more suitable.

## III. RESULTS

### A. Effect of the beam intensity and the number of diffraction patterns on the 3D diffraction intensity distribution under no angle estimation errors

**FIG. 3.**
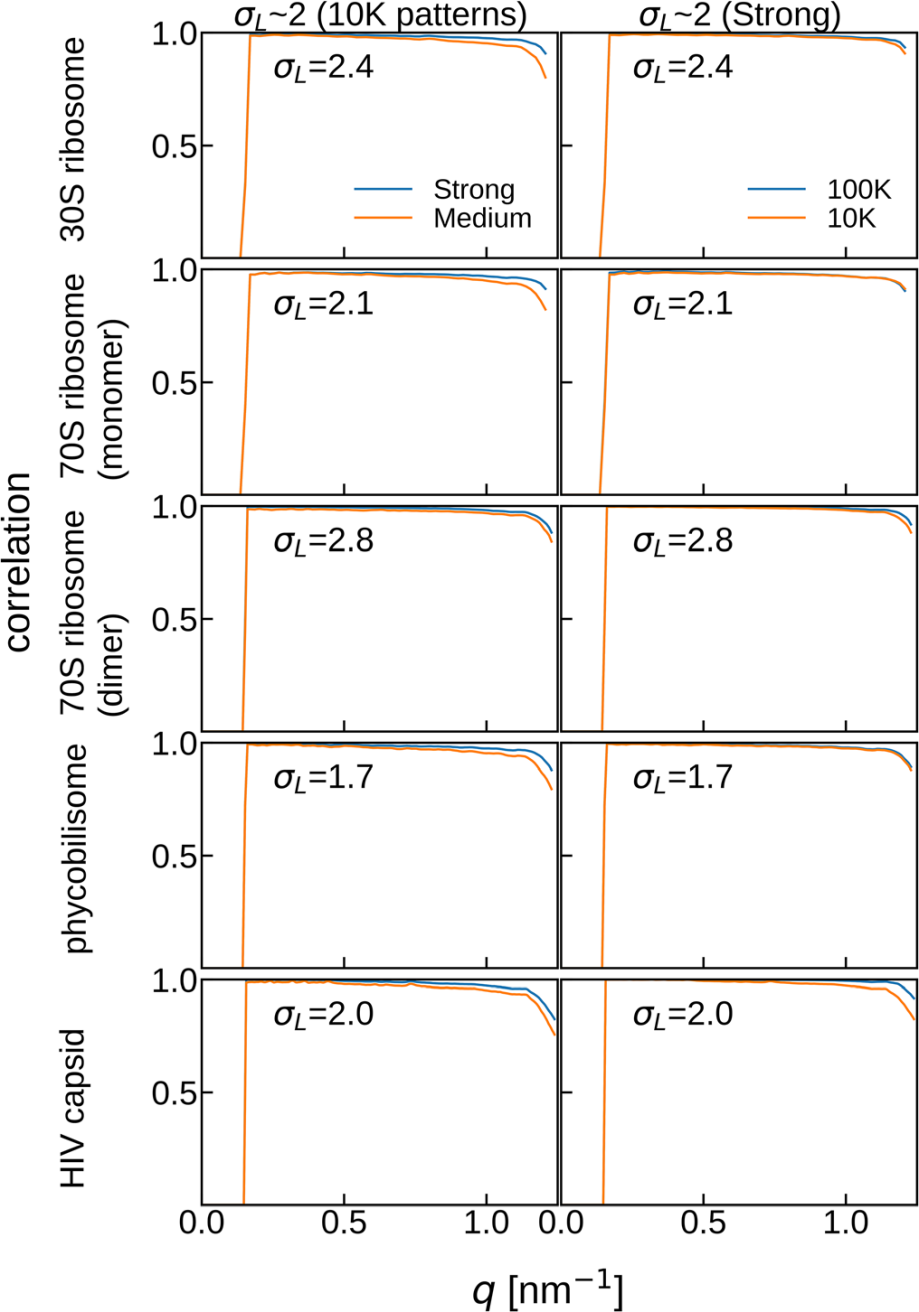
Spherical correlation between 3D diffraction intensity distributions *F_answer_* and *F_true_angle_. F_answer_* is the Fourier-transformed structure of the electron density map created from the PDB structure. *F_true_angle_* is reconstructed with diffraction patterns using *true* angles. (Left) Spherical correlations when *F_true_angle_* is reconstructed with 10 K patterns with σ_*L*_ ∼ 2 irradiated by strong and medium beam intensities. (Right) Spherical correlations when *F_true_angle_* is reconstructed with different numbers of diffraction patterns with σ_*L*_ ∼ 2 irradiated by the strong beam intensity. The diffraction patterns used here correspond to bin4 for 30S ribosome and 70S ribosome (monomer), bin2 for 70S ribosome (dimer) and phycobilisome, and bin1 for HIV capsid in Table I.

To assess the extent to which the 3D diffraction intensity distribution can be reproduced from 2D diffraction patterns in an ideal situation, the spherical correlations between *F_answer_* and *F_true_angle_*, reconstructed from the diffraction patterns with the binning factors corresponding to σ_*L*_ ∼ 2, were calculated (Fig. 3). Evidently, the spherical correlations slightly decreased for the lower beam intensity in the region *q* > 1.0 nm^−1^. The correlation also decreased when the number of diffraction patterns was low, particularly for larger molecules.

### B. Spherical average of photon counts for each diffraction simulation condition

The photon count in each voxel in the reconstructed 3D diffraction intensity distribution affects the signal-to-noise ratio and difficulty of phase retrieval. We calculated the spherical average of the photon counts per voxel in the 3D diffraction intensity distribution reconstructed with 2D diffraction patterns. This procedure is equivalent to calculating the radial average of the averaged 2D diffraction patterns.

**FIG. 4.**
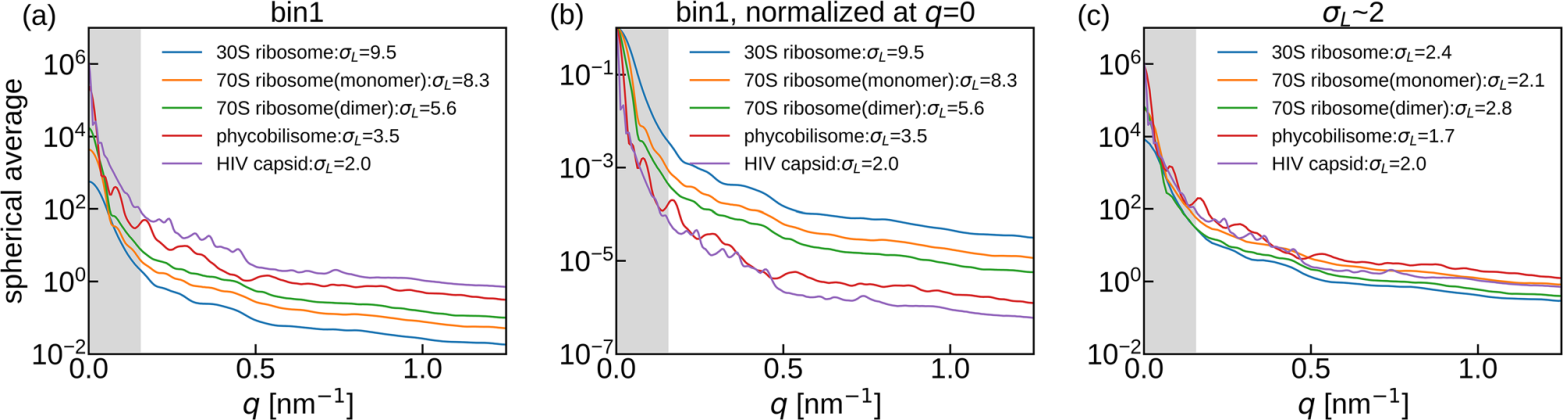
(a) Spherical average of photon counts per voxel in *F_true_angle_* reconstructed using the diffraction patterns without binning with strong beam intensity. (b) Spherical average profiles normalized at *q* = 0 of the data shown in (a). (c) Spherical average of photon counts per voxel in *F_true_angle_* reconstructed with σ_*L*_ ∼ 2 diffraction patterns with strong beam intensity. The diffraction patterns used here correspond to bin4 for 30S ribosome and 70S ribosome (monomer), bin2 for 70S ribosome (dimer) and phycobilisome, and bin1 for HIV capsid in Table I. The gray shadowed region is the area where masked by the beam stopper.

Figure 4(a) shows the spherical average of the 3D volume reconstructed with the unbinned patterns. Patterns from larger molecules contained more photons in each voxel at the same *q* compared with those from smaller molecules. The photon counts were normalized at *q* = 0, which revealed that the photon counts for larger molecules quickly decreased with increasing *q* compared with those for smaller molecules [Fig. 4(b)].

Among the simulated diffraction patterns used in this study, HIV capsid could use only unbinned diffraction patterns with σ_*L*_ = 2 to retrieve the phase information. To compare the photon counts per voxel for the same σ_*L*_ instead of the same pixel size, the photon counts per voxel in *F_true_angle_* with σ_*L*_ ∼ 2 were compared [Fig. 4(c)]. The differences among the molecules in photon counts have decreased, and the correlation with molecular size has been lost. Binned patterns (lower σ_*L*_) could also be used for smaller molecules, for which each pixel size was larger and had more photon counts.

### C. Angle estimation by slice matching

In the single-particle analysis using XFEL, the sample orientation captured on each diffraction pattern must be estimated to reconstruct the 3D diffraction intensity distribution. To estimate these angles, we previously developed slice matching algorithm (described below).^20^ The errors in the estimated angles were calculated to evaluate the slice matching performance for each condition.

First, the 3D diffraction intensity distribution, *F_true_angle_*, was reconstructed with 2D experimental diffraction patterns, *I_exp_*, using *true* angles (identical to the distribution examined in Sec. II B). This 3D distribution was used as the reference volume in the next step. Second, reference pattern library sets were created with 1° angular spacing from the reference volume. The library contained approximately 40 K reference patterns, *I_ref_*. Next, the best matched reference pattern $I_{\mathrm{ref}}^{\mathrm{best}}$ in the library was determined for each *I_exp_*. Finally, the angle difference between the *true* angle for each *I_exp_* and the angle for its best matched reference pattern, $I_{\mathrm{ref}}^{\mathrm{best}},$ was calculated. Although the binned pattern cannot be used for phase retrieval for larger molecules, it is still useful for angle estimation to reduce the calculation cost. Therefore, we calculated angle errors for all diffraction patterns, including the binned patterns with σ_*L*_ < 2.

**FIG. 5.**
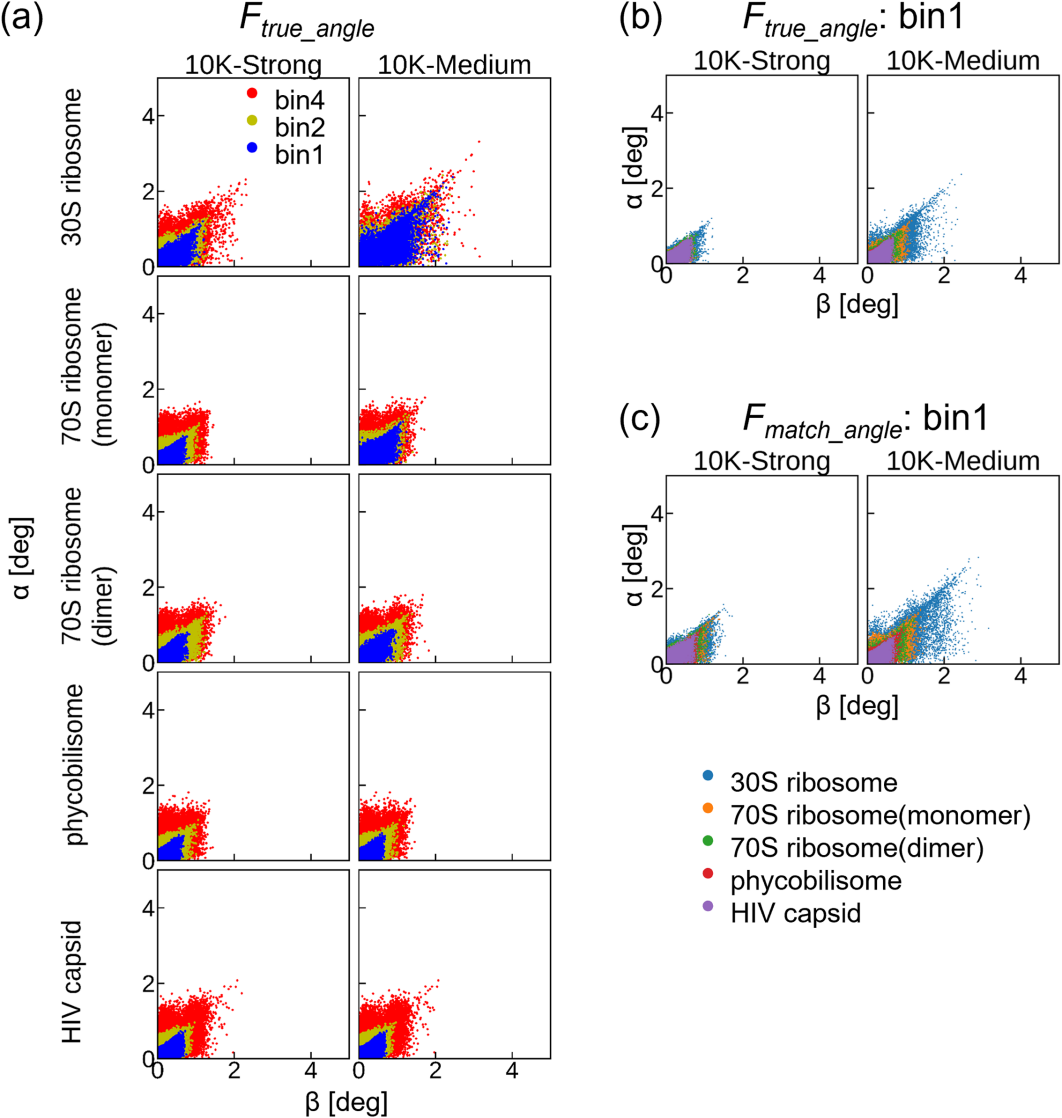
Angle errors by slice matching. α and β are the in-plane angle error and the angle between two planes, respectively. (a) Angle errors for slice matching against *F_true_angle_*, reconstructed with diffraction patterns using *true* angles. The labels bin1, bin2, and bin4 represent the binning factors of the diffraction patterns used for the creation of *F_true_angle_*. (b) Angle errors among the different molecules. The data are the same as bin1 in (a). (c) Angle errors among the different molecules for slice matching against *F_match_angle_*, reconstructed with diffraction patterns without binning using *matched* angles by slice matching. The dot colors are the same between (c) and (b).

Figure 5 shows the in-plane angle error, α, and the angle between the two planes, β. Our slice matching protocol estimated the angles correctly within the limits of the pixel sampling rate when the correct reference volume was provided [Fig. 5(a)]. In Fig. 5(b), the angle errors among the different molecules were compared when the unbinned diffraction patterns were used to reconstruct *F_true_angle_*. Evidently, the angle errors increased with decreasing molecular size. Thus, a finer speckle size and more scattered photons for larger molecules assist in an accurate angle search.

In an actual experimental situation, the correct reference volume is unknown. Thus, the accuracy of the angle estimation without a known suitable reference volume must be examined for practical applications. To estimate these angles, we performed the slice matching.^20^ The initial reference volume was generated from 2D diffraction patterns assigning random angles to each diffraction pattern. Angle estimation was performed with the same procedure, creating the reference pattern library sets and finding the best match. The reference volume was iteratively updated from 2D diffraction patterns by applying the estimated Euler angles. In the first stage of the slice matching iteration, the patterns with a binning factor of four were used to estimate the particle orientations. In the following stages, the angles were refined by modifying the binning factor to two. At the last stage of the iteration, the 3D diffraction intensity distribution, *F_match_angle_*, was reconstructed by applying the angles estimated by the patterns without binning. Finally, *F_match_angle_* was aligned to *F_true_angle_* for the angle error calculation.

Although the angle errors for *F_match_angle_*, shown in Fig. 5(c), were larger than those for *F_true_angle_*, shown in Fig. 5(b), they were less than 2°, except for 30S ribosome with medium beam intensity. We believe that these errors were sufficiently small because the angular spacing of *I_ref_* was 1°. However, these errors have nonnegligible effects on the final resolutions of the 3D structures as discussed later. Overall, the angle errors increased with a lower beam intensity or smaller molecular size, thus demonstrating that a sufficient photon count is important for accurate angle estimation.

### D. Accuracy of phase-retrieved molecular structure

To restore the molecular structure in real space, phase retrieval was performed using the hybrid input–output (HIO) algorithm combined with shrink-wrap.^41^

Phase retrieval for a 3D diffraction intensity distribution with a large number of voxels consumes a large amount of computational resources. To reduce the calculation time, phase retrieval was started from the 3D diffraction intensity distribution consisting of a small number of voxels. For small molecules, we started from 3D data reconstructed from binned patterns, still keeping σ_*L*_ > 2. For larger molecules, we started from the 3D data cropped the high-angle regions. The number of voxels was increased using the patterns with a higher oversampling ratio or by extending to the higher angle regions in the following steps. Using the results from the previous stage as the initial structure for the next stage, the iteration number of the phase retrieval could be reduced, and stable results were obtained, rather than starting directly with a volume consisting of many voxels. Table II lists the phase retrieval procedures for each molecule and the number of voxels in the 3D diffraction intensity distributions.

**TABLE II.** Procedures of phase retrieval.

Molecule	Phase retrieval step	Binning factor	Label	Number of voxels	Radius of 3D diffraction intensity distribution		Real space voxel size
					(nm^–1^)	(nm)	(nm)
30S ribosome 70S ribosome (monomer)	1	4	bin4	147 × 147 × 147	1.25	(0.8)	0.4
	2	2	bin2	295 × 295 × 295	1.25	(0.8)	0.4
	3	1	bin1	591 × 591 × 591	1.25	(0.8)	0.4
70S ribosome (dimer) phycobilisome	1	2	⋯	147 × 147 × 147	0.625	(1.6)	0.8
	2	2	bin2	295 × 295 × 295	1.25	(0.8)	0.4
	3	1	bin1	591 × 591 × 591	1.25	(0.8)	0.4
HIV capsid	1	1	⋯	147 × 147 × 147	0.3125	(3.2)	1.6
	2	1	⋯	295 × 295 × 295	0.625	(1.6)	0.8
	3	1	bin1	591 × 591 × 591	1.25	(0.8)	0.4

Herein, 40 phase retrievals were performed for each 3D diffraction intensity distribution, starting from different initial phases; further, the restored structures were averaged. For HIV capsid, 80 phase retrievals were performed because the convergence of phase retrieval transfer functions (PRTFs)^3,42^ was worse (Figs. S1 and S2). These restored structures were labeled as bin4, bin2, and bin1, representing the binning factors of the diffraction patterns used to reconstruct the 3D diffraction intensity distribution, which was used for phase retrieval. Finally, the restored molecular structure with a 0.4-nm sampling rate was compared in real space.

To quantify how well phase information was retrieved, the phase retrieval transfer functions (PRTFs) were calculated among the phase retrieval trials, starting from the different initial phases. PRTF shows confidence in the retrieved phases as a function of the spatial frequency.^3^ Additionally, we calculated Fourier shell correlations (FSC) between the average of the restored structures and the original electron density map created from PDB structures. The FSC measures the normalized cross correlation coefficient between two structures in real space over the corresponding shells in reciprocal space.^43^ In the current standard protocol for single-particle analysis, FSC is calculated between the volumes restored by two independent halves of the dataset (gold-standard FSC).^12,44^ Because the molecular structures were known in this study, FSC was used herein to measure how close the restored structure was to the *true* structure.

**FIG. 6.**
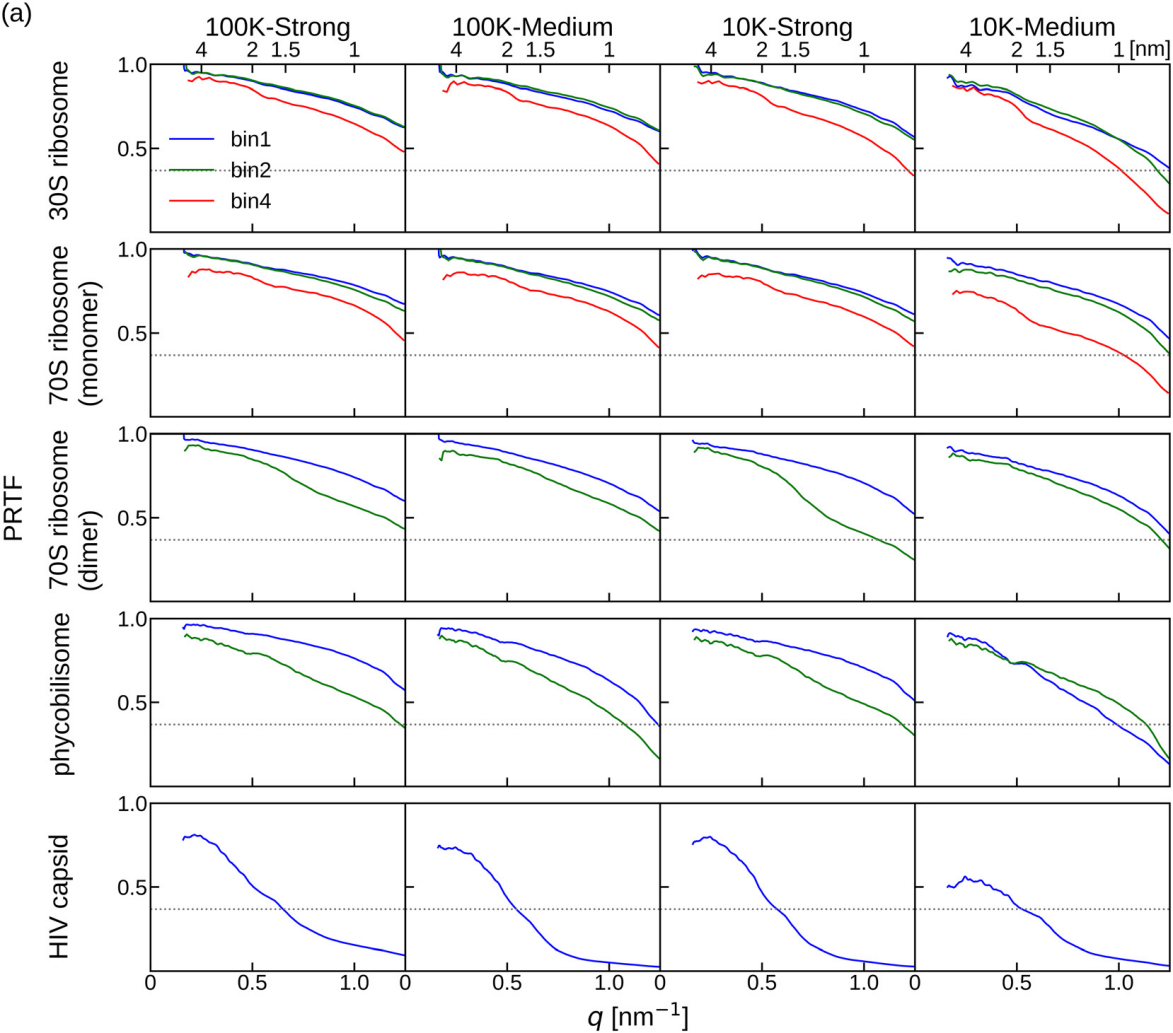

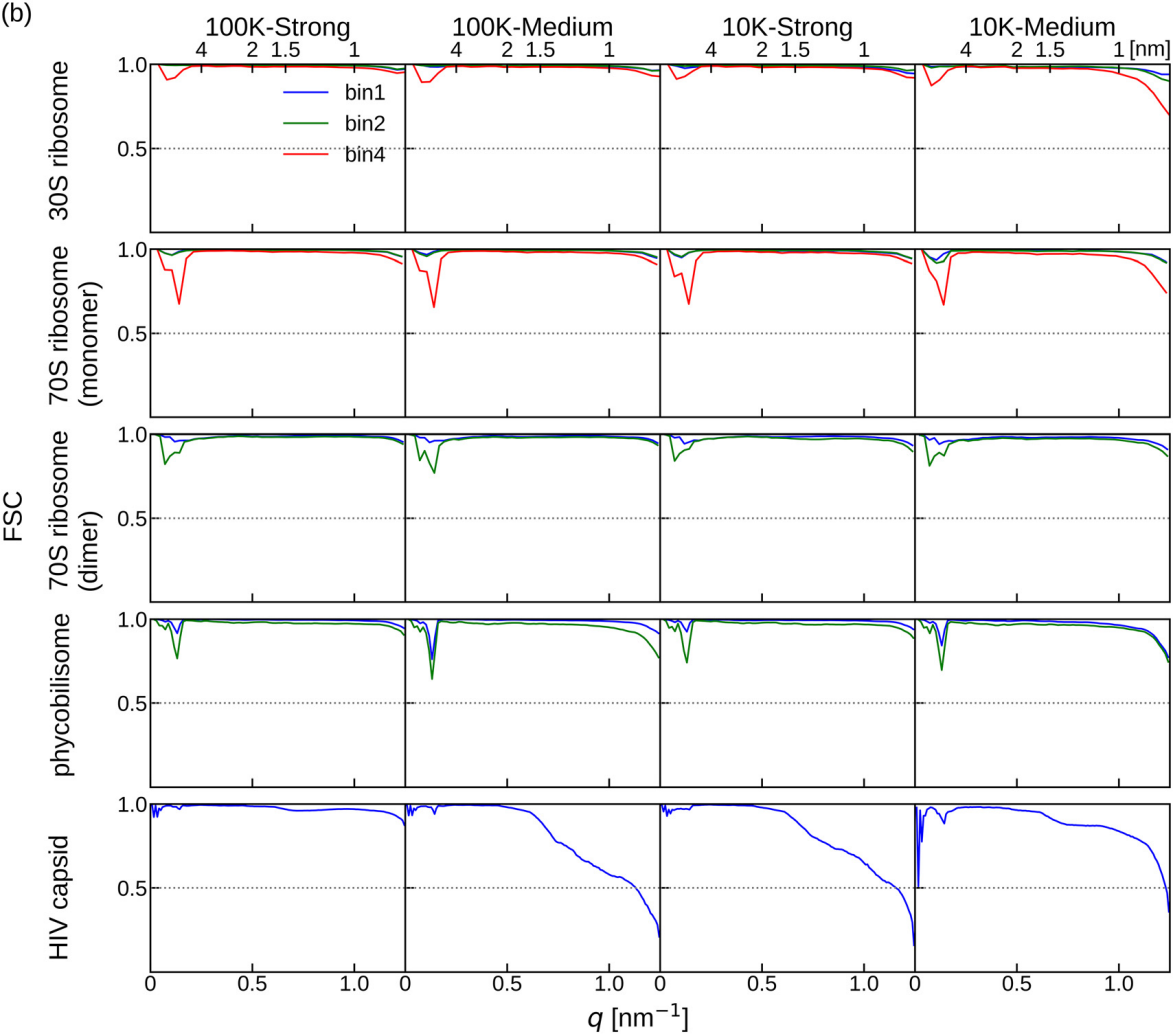
(a) PRTF among the phase retrieval trials from *F_true_angle_* reconstructed using *true* angles with diffraction patterns. Dotted line is the PRTF threshold value, 1/e. (b) FSC between the PDB structure and the averaged molecular structure restored from *F_true_angle_*. The labels bin1, bin2, and bin4 represent the binning factors of the diffraction patterns used to reconstruct *F_true_angle_.*

Figure 6 shows the PRTF and FSC curves for the phase retrieval results from *F_true_angle_* reconstructed using *true* angles. The large decrease below 0.2 nm^−1^ that appeared on the FSC curves reflected the lack of information by the mask mimicking the beam stopper. A comparison of the PRTF curves among the tested molecules revealed that the reconstruction was at a lower resolution for larger molecules. PRTF curves for HIV capsid were worse than those for other molecules, particularly for 10 K patterns with medium beam intensity. Similarly, the FSC curves for HIV capsid were worse than those for other molecules. Except for HIV capsid, the FSC exhibited high values to the detector limit (1.25 nm^−1^). The reason behind the difficulty in the restoration of HIV capsid structure is discussed later.

The PRTF and FSC values were low for the results of small oversampling ratios (more binning) for each molecule (Table I lists σ_*L*_ and binning factors). However, for 30S ribosome and 70S ribosome (monomer), the results were almost the same with σ_*L*_ > 4. These results are consistent with the previous study that an oversampling ratio above four is required for accurate phase retrieval.^34,38^

**FIG. 7.**
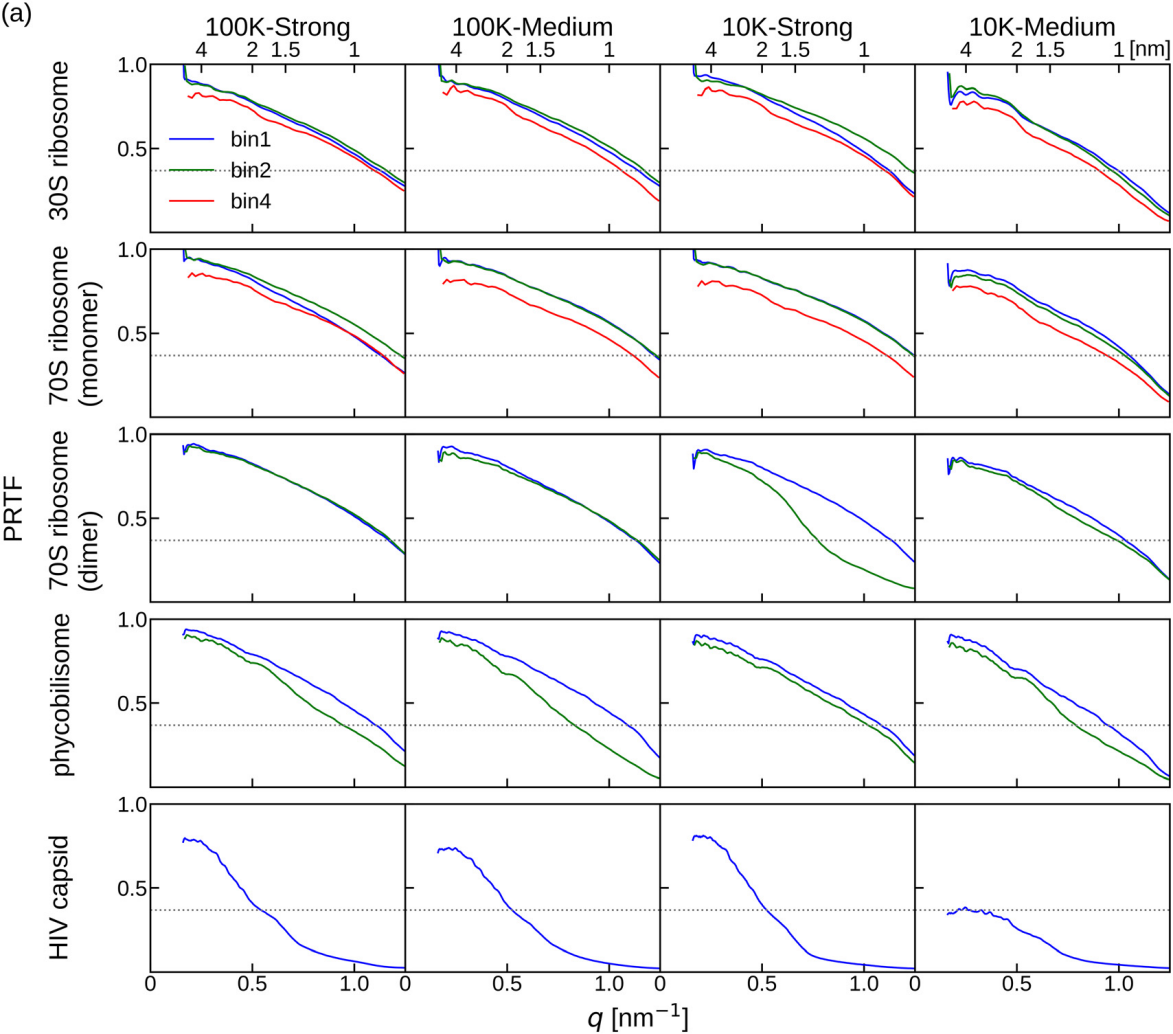

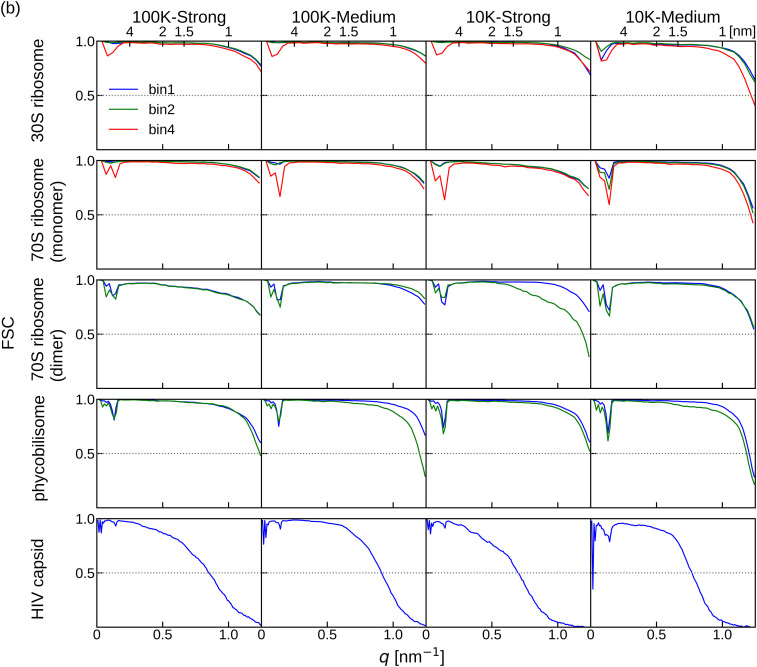
(a) PRTF among the phase retrieval trials from *F_match_angle_* reconstructed using *matched* angles with diffraction patterns. Dotted line is the PRTF threshold value, 1/e. (b) FSC between the PDB structure and the averaged molecular structure restored from *F_match_angle_*. The labels bin1, bin2, and bin4 represent the binning factors of the diffraction patterns used to reconstruct *F_match_angle_.*

In the same manner, the PRTF and FSC curves for the phase retrieval results from *F_match_angle_* reconstructed using the angles estimated by slice matching protocol were examined (Fig. 7). Both the PRTF and FSC values were lower than the results for *F_true_angle_*. These results were expected because *F_match_angle_* was reconstructed with the *matched* angles, which included some errors. However, the trends were similar to those of *F_true_angle_*. The PRTF curves indicated that the reconstruction was at a lower resolution for larger molecules [Fig. 7(a)]. The FSC values at the detector edge resolution were still higher than 0.5, except for HIV capsid [Fig. 7(b)].

Table III summarizes the resolutions at PRTF = 1/e for the restored molecular structures. For the structures restored from *F_true_angle_*, the PRTF resolutions were almost the same except for HIV capsid. For the structures restored from *F_match_angle_*, there are noticeable molecular size effects on the PRTF resolution, which worsens with increasing molecular size. For the reconstruction using the patterns with medium beam intensity, the use of 100 K patterns consistently improved the resolution compared to 10 K patterns. The resolutions obtained by 10 K-strong and 100 K-medium are similar in accordance with the previous theory.^24^ On the other hand, by increasing the diffraction patterns from 10 to 100 K for the strong beam, the achieved resolutions were not improved drastically. These results indicate that increasing the number of diffraction patterns is especially beneficial in reducing the signal-to-noise ratio for weak patterns.

**TABLE III.** (a) Resolution in nm at PRTF = 1/e for the molecular structures restored from *F_true_angle_* reconstructed using *true* angles with diffraction patterns. (b) Resolution in nm at PRTF = 1/e for the molecular structures restored from *F_match_angle_* reconstructed using *matched* angles with diffraction patterns.

(a)					
Molecule	σ_*L*_	100 K-strong	100 K-medium	10 K-strong	10 K-medium
30S ribosome	9.5	0.80	0.80	0.80	0.80
	4.8	0.80	0.80	0.80	0.84
	2.4	0.81	0.81	0.82	0.98
70S ribosome (monomer)	8.3	0.80	0.80	0.80	0.80
	4.2	0.80	0.80	0.80	0.80
	2.1	0.81	0.81	0.81	0.98
70S ribosome (dimer)	5.6	0.80	0.80	0.80	0.80
	2.8	0.80	0.80	0.93	0.83
Phycobilisome	3.5	0.80	0.81	0.80	1.01
	1.7	0.81	0.93	0.84	0.88
HIV capsid	2.0	1.54	1.83	1.74	1.91
(b)					
30S ribosome	9.5	0.89	0.88	0.89	1.00
	4.8	0.86	0.86	0.81	1.04
	2.4	0.90	0.95	0.90	1.11
70S ribosome (monomer)	8.3	0.89	0.82	0.80	0.95
	4.2	0.81	0.81	0.81	0.98
	2.1	0.88	0.89	0.90	1.07
70S ribosome (dimer)	5.6	0.86	0.89	0.88	0.97
	2.8	0.85	0.88	1.30	1.01
Phycobilisome	3.5	0.91	0.92	0.92	1.06
	1.7	1.06	1.20	0.98	1.27
HIV capsid	2.0	1.85	1.91	1.91	3.11

**FIG. 8.**
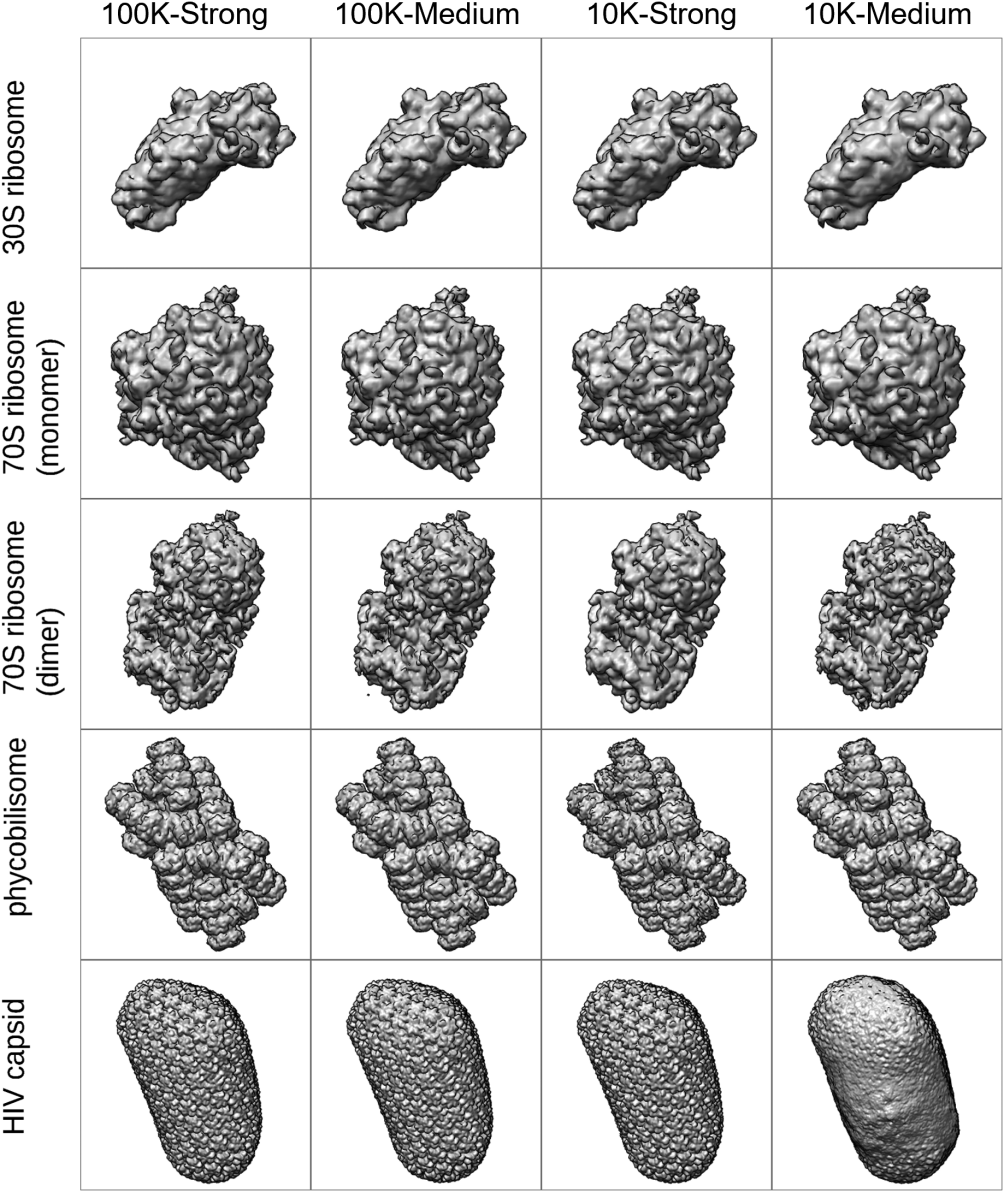
Averaged molecular structures restored from *F_match_angle_* reconstructed using the diffraction patterns with σ_*L*_ ∼ 2.

The average restored molecular structures from *F_match_angle_* reconstructed using the diffraction patterns with σ_*L*_ ∼ 2 are shown in Fig. 8. All molecular structures were well restored, except for HIV capsid with 10 K diffraction patterns irradiated by the medium beam intensity (as shown in the bottom right of Fig. 8). This poorly restored appearance reflects the lower PRTF values, and the deep and narrow dimples at the very small *q* value on the FSC curve shown in Fig. 7 represented the difficulty of phase retrieval. The high FSC values in the large *q* region of this FSC curve may be owing to the artifact of the blurred diffraction patterns caused by the small number of scattered photons.^45^ Other 3D structures of HIV capsid were well restored, even though their FSC curves were noticeably lower than those of other molecules. This is discussed in Sec. IV.

The results of the slice matching are based on the initial random angle set. Therefore, the slice matching calculations were repeated, starting from different random angle sets, to confirm the above observations (Figs. S3 and S4). Slice matching results from different random angle sets exhibited consistent behavior between all molecules under all conditions except for the FSC curve for HIV capsid with 10 K diffraction patterns with medium beam intensity. The low-FSC curve at the bottom right of Fig. S3(b) well reflects the poor appearance of the corresponding molecular structure shown in Fig. S4. The artifacts by the small number of scatter photons shown in Fig. 7(b) did not appear in Fig. S3(b).

## IV. DISCUSSION AND CONCLUSION

Biomolecules larger than 100 nm in thickness can be considered as suitable targets for single-particle analysis using XFELs. Owing to the high transmittance of x-rays, the inner structure of such thick molecules can be observed without multiple scattering, which is not feasible using electron microscopy. A large x-ray cross section of large molecules can assist in restoring the molecular structure with stronger signals. However, the resolution of the restored molecular structure decreases with increasing molecular size. The possible reasons for this low resolution are inaccurate angle estimation, the insufficiency of the number of diffraction patterns to cover the 3D diffraction intensity distribution, and insufficient photon counts. Therefore, we will discuss each contribution.

First, the angle estimation errors decreased with increasing molecular size (Fig. 5). Walczak and Grubmüller demonstrated that increasing the number of photons improves the accuracy of orientation estimation.^46^ Since the pixels of the same size captured more photons from larger molecules than smaller ones, as shown in Fig. 4(a), larger molecules could reduce the angular error. The finer speckle size of the larger molecule also improved the angle estimation accuracy.

By contrast, the PRTF and FSC curves tended to be lower for larger molecules, even when the 3D diffraction intensity distributions were reconstructed using *true* angles (Fig. 6). Moreover, the differences between the PRTF or FSC of the phase recoveries from *F_true_angle_* and *F_match_angle_* (Figs. 6 and 7) were more significant for larger molecules. These results indicate that while the angle estimation was sufficiently accurate, the inaccuracies within the 3D diffraction intensity distribution were more sensitively reflected in the results of phase retrieval for larger molecules.

**FIG. 9.**
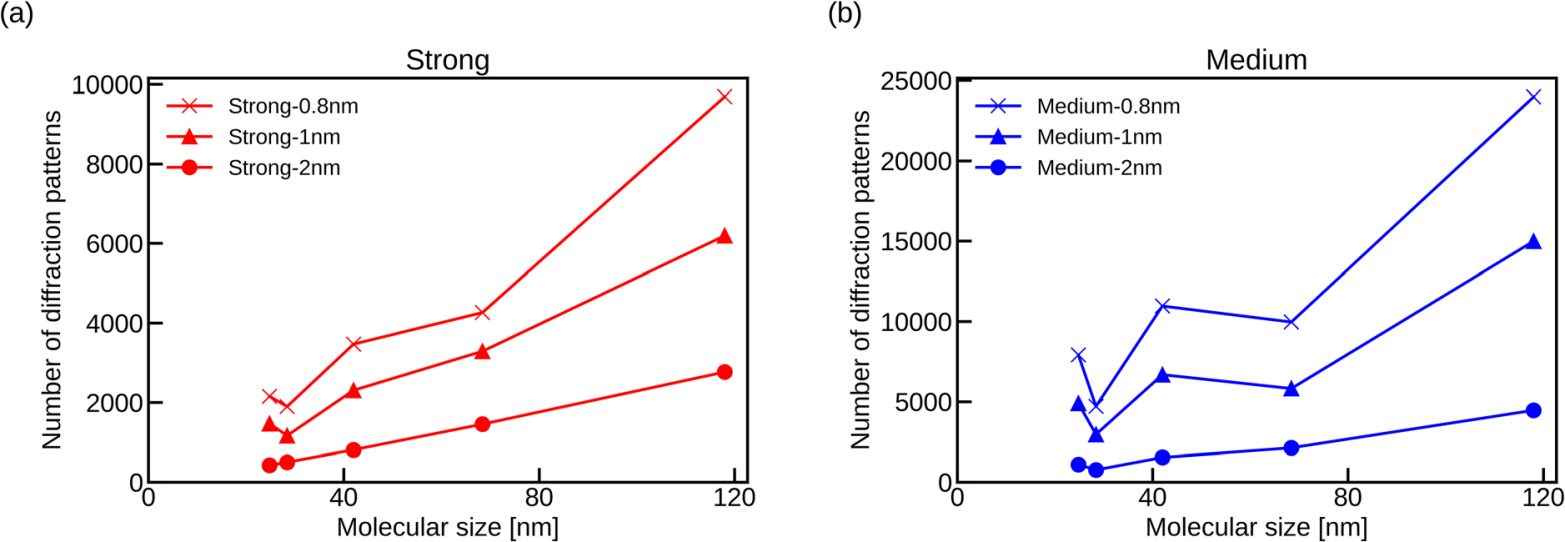
Required number of diffraction patterns for the target resolution against the molecular size for (a) strong beam intensity and (b) medium beam intensity.

With regard to the number of diffraction patterns required, some previous studies have shown that a larger molecule requires numerous diffraction patterns for reconstructing its 3D structure.^47,48^ Following the procedure proposed in Poudyal's study,^24^ we estimated the number of diffraction patterns required to achieve some target resolutions from the average number of scattered photons in the pixels with the condition σ_*L*_ ∼ 2 from our simulations [Fig. 4(c)]. Naturally, more patterns are necessary to achieve higher resolution. Also, the required number of diffraction patterns is generally higher with a larger molecular size (Fig. 9). However, the order is not exact; between phycobilisome and ribosome 70S (dimer) and between 30S ribosome and 70S ribosome (monomer) are switched. This is because photon counts for phycobilisome and ribosome 70S (monomer) were larger than others when counted for the pixels with σ_*L*_ ∼ 2, due to the variations of mass distributions, which reduces the required number of diffraction patterns. This effect was more significant for medium beam intensity.

Using the Poudyal theory, we also estimated the expected resolution using the 10 K-medium diffraction pattern set (Fig. S5). The expected resolutions were 0.78, 0.72, 0.84, 0.8, and 1.3 in nm for 30S ribosome, 70S ribosome (monomer), 70S ribosome (dimer), phycobilisome, and HIV capsid, respectively. Our achieved FSC resolutions with threshold = 0.5 on the most right column of Fig. 7(b) were <0.80 nm for ribosomes, 0.83 nm for phycobilisome, and 1.3 nm for HIV capsid. FSC resolution for HIV capsid for 10 K-medium diffraction patterns in Fig. 7(b) might be overestimated due to the artifact by too small captured photons.^45^ In our numerical simulation, while expected resolutions are achieved for small molecules, they are lower for large ones. One possible reason is the nonuniformity of the molecule, which is not considered by Poudyal's study. HIV capsid has the characteristic shape having a large internal cavity. Its photon scattering ability might be less compared to its size. Other possible reasons are the limitation of our numerical algorithms, including the process for the reconstruction of 3D diffraction intensity distribution, such as angle estimations and interpolation, and phase retrieval.

Figure 9 also shows that to achieve the diffraction edge limited resolution, 0.8 nm and 30 K diffraction patterns were enough. We used 40 K reference patterns for slice matching. More experimental diffraction patterns could reduce the shot noise, but using more reference patterns would not be effective for the accurate angle estimation but only increases the computation time.

The choice of binning also affects the outcome of the 3D reconstructions. Molecular size influences the choice of binning of diffraction patterns and, in turn, the oversampling ratio; the effects appeared more clearly in the phase recovery process. The PRTF and FSC curves with σ_*L*_ ∼ 2 were worse than 4 for all molecules regardless of the number of diffraction patterns and beam intensity, whereas the results with σ_*L*_ > 4 were almost the same (Figs. 6, 7, and S3). These results were consistent with our previous study, which showed that σ_*L*_ ∼ 4 was sufficient to retrieve the phase information for a 3D structure.^34^ Therefore, diffraction patterns with an oversampling ratio of σ_*L*_ ∼ 4 would provide good reconstruction, but increasing the oversampling ratio for larger molecules is challenging in two aspects.

In practice, the choice of oversampling ratio would be determined by the molecular size. To maintain the same oversampling ratio, diffraction patterns for larger molecules should consist of smaller pixel sizes in the unit of spatial frequency. For example, the pixels in the diffraction pattern of bin1 for HIV capsid (σ_*L*_ = 2.0) were finer than those of bin4 for ribosome 30S (σ_*L*_ = 2.4) (see Fig. 2). Consequently, the number of voxels in the 3D diffraction intensity was higher; thus, more diffraction patterns were required to reconstruct the 3D map accurately. In addition, the computational cost of handling a 3D structure consisting of numerous voxels could grow rapidly; therefore, in this study, for HIV capsid, only the lowest value of σ_*L*_ = 2 could be examined. Thus, although larger molecules have larger cross sections to x-rays, utilization of this advantage may not be straightforward.

In our results, HIV capsid was a difficult target where we could obtain the structures only at lower resolution. Because the external shapes of large molecules depend mainly on the lower *q* region in reciprocal space, the information lost due to the beam stopper can be crucial. Since the mask radius we simulated was rather large, especially for HIV capsid, we analyzed the achievable resolution of the HIV capsid by reducing the size of the center mask to 0.034 nm^−1^ (Fig. S6). Even reducing the mask size, absolute resolutions were still lower than those for other molecules, although PRTF profiles at low *q* region were improved, especially for the 10 K-medium diffraction pattern set. The beam stopper size affects the confidence of phase retrieval at low *q*-region but could not improve the restored molecules resolution.

**FIG. 10.**
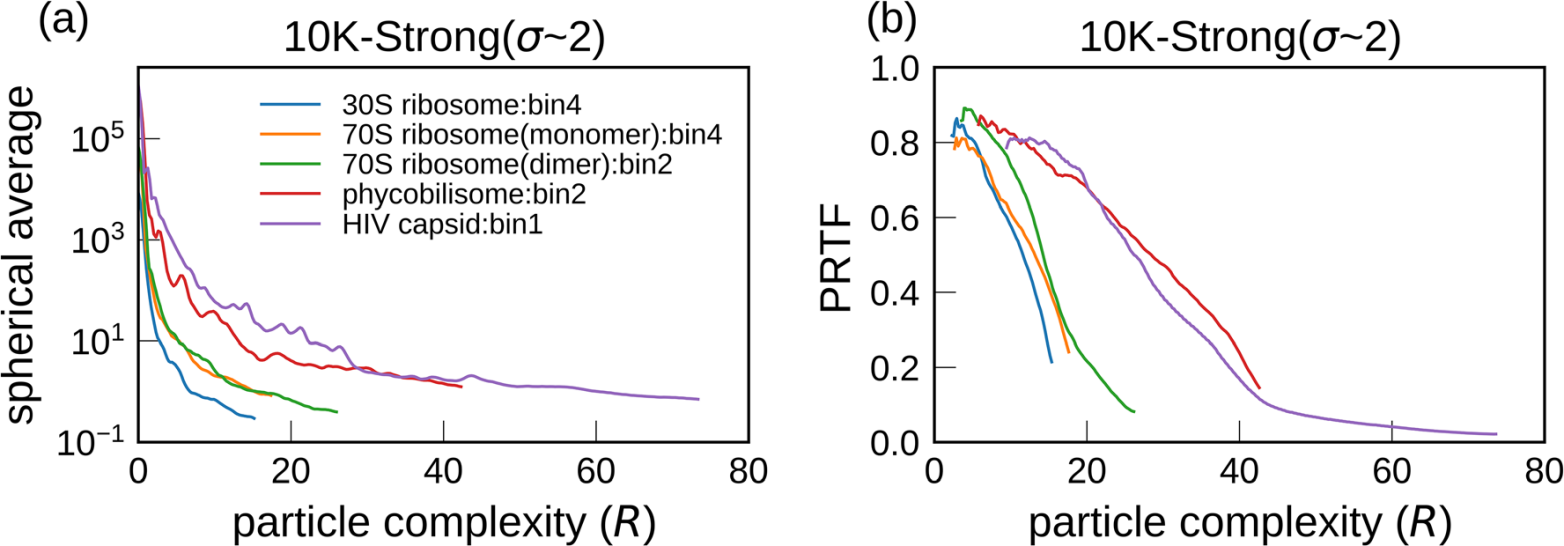
(a) Spherical average of photon counts per voxel in *F_true_angle_*, against the particle complexity, *R*. (b) PRTF against particle complexity, *R*, among the phase retrieval trials from *F_match_angle_* restored from 10 K diffraction patterns with σ_*L*_ ∼ 2 irradiated by strong beam intensity. These figures replotted the results in Fig. 4(c) and Fig. 7(a) against *R* instead of *q.*

While our simulations revealed that the achievable resolution is generally lower for larger molecules, another aspect is how much detail of the structure can be restored with respect to the molecular size. Therefore, we consider another measure, particle complexity, *R*, which is a nondimensional parameter defined as the particle radius divided by the resolution.^47,48^ We consider *R*, defined as (*L*/2)/(1/*q*), where *L* denotes the molecular size and *q* denotes the spatial frequency. Using this measure, we replotted the spherical averages of photon counts per voxel in Fig. 4(c) and PRTF curves in Fig. 7(a) against *R* instead of *q* (Fig. 10). For this assessment, PRTF was used instead of FSC because the FSC curves shown in Fig. 7(b) were high at the detector edge. The average photon counts increased with molecular size in the same particle complexity region [Fig 10(a)]. Using the same PRTF criteria, the complexity level achieved was higher for larger molecules than for smaller ones [Fig. 10(b)]. Visually, the reconstructed 3D structures of larger molecules were of good quality because they were recovered at a higher particle complexity; however, their absolute resolution was lower.

This study only considered the Poisson noise to the simulated diffraction patterns. In the actual experimental data, many background noises should be considered.^37,49^ Although the binning process could reduce the shot noise, it is not effective to reduce other background noises. In addition, some background noises originate from sample conditions, such as aggregation, hydration, and contamination. These noise contributions differ for each molecule and may not simply relate to the molecular size. A more realistic simulation will be required for further accurate estimation according to each target molecule. Nonetheless, we believe our simulations should provide valuable data for selecting the target molecular size for experiments.

In conclusion, using XFEL single-particle analysis, high-resolution reconstruction of larger molecules with high resolution is challenging, even though their diffraction powers are high. This is because the high-resolution structural reconstruction of large molecules requires more detailed 3D diffraction intensity distributions from a larger number of patterns with fine pixel resolutions. More improvements in beamline and pixel photon detectors would be beneficial to achieve a strong beam intensity to bring enough photon counts to the fine pixel size. In addition, further improvement of the reconstruction algorithms to take full advantage of the available data is necessary. Such molecular size dependency can also be understood by considering particle complexity; large molecules can be resolved at higher particle complexity relative to the molecular size compared with small molecules. To study the structures of larger molecules at high resolution with XFEL, hybrid approaches combining information obtained from different experiments and computational modeling would be helpful.^50–52^ Combining these techniques can improve structural reliability and provide new insights into molecular conformational dynamics.

## SUPPLEMENTARY MATERIAL

See the supplementary material for further details of reconstruction parameters and additional data.

## Data Availability

The data that support the findings of this study are available from the corresponding author upon reasonable request. The codes for slice matching used in this study are openly available at https://riken-share.ent.box.com/s/eobvldhkul31rv965bvzhvofs3yuaca2, Ref. 53.
